# Patterns of disparity: age and socioeconomic differences in women’s smoking and quitting outcomes in Great Britain

**DOI:** 10.1186/s12916-025-04574-1

**Published:** 2026-02-10

**Authors:** Sarah E. Jackson, Caitlin Notley, Sharon Cox

**Affiliations:** 1 Department of Behavioural Science and Health, University College London, 1-19 Torrington Place, London, WC1E 7HB UK; 2 Faculty of Medicine and Health Sciences, Norwich Medical School, University of East Anglia, Norwich, UK; 3 Behavioural Research UK, Edinburgh, UK

**Keywords:** Women, Sex, Gender, Age, Life stage, Smoking, Smoking cessation

## Abstract

**Background:**

Smoking poses health risks to women across the lifespan. This study aimed to examine age-related differences in smoking, quit attempts, and cessation outcomes among women in Great Britain, overall and by socioeconomic position.

**Methods:**

We analysed cross-sectional data from 30,519 women (≥ 16 years) in Great Britain participating in a nationally representative survey between 2023 and 2025. We used logistic regression with restricted cubic splines to obtain age-specific estimates of smoking prevalence, the quit attempt rate, the success rate of quit attempts, and the overall quit rate, among all women and by socioeconomic position (indexed by occupational social grade; ABC1 = more advantaged, C2DE = less advantaged). We calculated prevalence ratios (PR; C2DE/ABC1) to illustrate the extent of socioeconomic disparities.

**Results:**

Overall, smoking prevalence was highest among women in their 20s and 30s and declined with age. However, there were notable differences by socioeconomic position. While it declined steadily with age among more advantaged women, smoking prevalence peaked in the early 40s among less advantaged women and was more than twice that of more advantaged women in mid-life (PR range = 2.02–2.47 between ages 35 and 60). Quit attempts decreased linearly with age, with similar prevalence and trends across socioeconomic groups. The success rate of quit attempts was highest among women in their 20s and 30s, but dropped in mid-life and further in older age. Women from less advantaged backgrounds had lower success rates, particularly between ages 45 and 60 (PR range = 0.70–0.73). The overall quit rate among past-year smokers was highest at age 31 for more advantaged women (23.3%) and at age 25 for less advantaged women (22.9%). Quit rates were substantially lower between ages 40 and 60 among less advantaged women (PR range = 0.65–0.69).

**Conclusions:**

Smoking behaviours and cessation outcomes among women in Great Britain vary by both age and socioeconomic position, with particularly high smoking prevalence and low quit rates among less advantaged women in mid-life, corresponding with perimenopause and the menopausal transition. These disparities highlight the need for tailored smoking cessation strategies to improve quit success and reduce smoking prevalence across the lifespan.

**Supplementary Information:**

The online version contains supplementary material available at 10.1186/s12916-025-04574-1.

## Background

Smoking is a significant public health issue that poses unique risks for women across the lifespan, affecting both their immediate and long-term health. Women may be more susceptible than men to the impact of smoking cardiovascular disease [1] and chronic obstructive pulmonary disease [2] risk. Additionally, women who smoke face an increased risk of cervical cancer [3], osteoporosis [4], reduced fertility [5], and earlier onset of menopause [6]. For those women who become pregnant and smoke, there are heightened risks to both mother and the baby, such as preterm birth and infant low birth weight [7]. This underscores the need for a deeper understanding of smoking behaviours among women. While overall smoking rates in Great Britain have declined in recent decades [8], patterns of smoking and quitting vary by age [8–11] and gender [12], likely due to differences in social influences, cultural norms, health concerns, and life circumstances. However, the extent to which age influences smoking and quitting behaviours among women in Great Britain remains underexplored. Understanding this could inform tailored interventions to accelerate reductions in smoking prevalence among women.

Age-related differences in smoking behaviours can be shaped by various factors. Younger women may be more susceptible to peer norms including social pressures, and the glamourisation of smoking within the media and ideas perpetuated by historic images and tobacco marketing. Across cultural eras, tobacco smoking has been positioned as a gendered behaviour [13, 14]. The tobacco industry has targeted women with marketing strategies linking smoking with female empowerment, glamour, and thinness [15, 16]. Evidence suggests that younger women and new mothers in particular may be dissuaded from quitting smoking for fear of gaining weight [17–20]. As women progress through different life stages—such as pregnancy, parenting, or menopause—health concerns and hormonal changes may either increase or decrease their motivation to quit [21, 22]. Additionally, women may face unique challenges in quitting during certain periods of life due to factors like work-related stress, caregiving responsibilities (for children and/or parents), or financial pressures [23–25]. Generational differences in exposure to tobacco control policies, such as smoking bans and public health campaigns, may also influence smoking behaviours across age groups [26]. Furthermore, as women who smoke reach older age, they may feel less inclined to quit if they perceive that smoking has not harmed them so far or that the damage is already done [27]. Longer smoking durations also tend to be associated with higher levels of addiction and habit forming (especially among those who started younger), which may make quitting more difficult [28, 29].

Smoking behaviours and cessation outcomes are also driven by social inequalities. Socioeconomic position plays a key role in smoking prevalence, the likelihood of quitting, and susceptibility to relapse [8, 30, 31]. Women from less advantaged socioeconomic groups are more likely to smoke and face greater barriers to cessation, including financial stress and greater exposure to smoking in their social environments [30]. These disparities highlight the need to examine not only overall age-related differences in women’s smoking and quitting behaviours but also how these patterns vary by socioeconomic position. Understanding these age- and socioeconomic-related variations can help tailor smoking cessation strategies to the specific needs of women at different stages of life as well as across the social gradient.

This study aimed to examine age-related differences in smoking prevalence, quit attempts, and cessation outcomes in a nationally representative sample of women in Great Britain, both overall and by socioeconomic position.

## Methods

### Pre-registration

The study protocol and analysis plan were pre-registered on Open Science Framework (https://osf.io/w2smk).

### Design

Data were drawn from the Smoking Toolkit Study, an ongoing monthly cross-sectional survey of a representative sample of adults (≥ 16 years) in Great Britain [32, 33]. The study uses a hybrid of random probability and simple quota sampling to select a new sample of approximately 2450 adults each month. Data are collected through telephone interviews. Comparisons with other national surveys and sales data indicate the survey achieves nationally representative estimates of key variables such as sociodemographic characteristics, smoking prevalence, and cigarette consumption [32, 34].

The present analyses focused on data from women who responded to the survey between January 2023 and February 2025. This period was selected to provide up-to-date estimates while ensuring adequate sample size for analyses.

### Measures

Smoking status was assessed by asking participants which of the following best applied to them: (a) I smoke cigarettes (including hand-rolled) every day; (b) I smoke cigarettes (including hand-rolled), but not every day; (c) I do not smoke cigarettes at all, but I do smoke tobacco of some kind (e.g. pipe, cigar, or shisha); (d) I have stopped smoking completely in the last year; (e) I stopped smoking completely more than a year ago; (f) I have never been a smoker (i.e. smoked for a year or more). Responses a–c were considered current smoking.

Quit attempts were assessed among women who had smoked in the past year with the question: ‘How many serious attempts to stop smoking have you made in the last 12 months? By serious attempt I mean you decided that you would try to make sure you never smoked again. Please include any attempt that you are currently making and please include any successful attempt made within the last year’. Those who reported making at least one serious quit attempt in the past year were coded 1, else they were coded 0.

Success of quit attempts (i.e. quits among those who made an attempt) was assessed among women who tried to stop smoking in the past year with the question: ‘How long did your most recent serious quit attempt last before you went back to smoking?’ Those who responded that they were still not smoking will be coded 1, else they were coded 0.

Smoking cessation (i.e. overall quits) was assessed among all women who smoked in the past year using the question assessing smoking status. Those who responded ‘I have stopped smoking completely in the last year’ were coded 1 and those who reported current smoking were coded 0.

Age was analysed as a continuous variable, using restricted cubic splines (see Statistical analysis section). We also provided descriptive data in age bands (16–19 years, 5-year age bands from 20–24 years through 75–79 years, and ≥ 80 years).

Socioeconomic position was assessed with an occupational measure of social grade [35] and categorised as ABC1 (includes managerial, professional, and upper supervisory occupations) and C2DE (includes manual routine, semi-routine, lower supervisory, state pension, and long-term unemployed). This occupational measure of social grade is a valid index of socioeconomic position that is widely used in research in UK populations. It has been identified as particularly relevant in the context of smoking and quit attempts and success [36].

### Statistical analysis

Data were analysed using R v.4.4.1. The Smoking Toolkit Study uses raking to weight the sample to match the population in Great Britain. This profile is determined each month by combining data from the UK Census, the Office for National Statistics mid-year estimates, and the annual National Readership Survey [32]. The following analyses used weighted data. We excluded participants who did not report their age or smoking status. Missing data on other variables were excluded on a per-analysis basis.

We estimated the prevalence of each outcome, among women of all ages, overall and by socioeconomic position. To quantify the extent of any differences between women who were more and less advantaged, we reported these estimates alongside prevalence ratios (PRs; calculated as prevalence among less advantaged women divided by prevalence among more advantaged women) and 95% confidence intervals (CIs) calculated using bootstrapping (1000 replications).

Age-specific estimates of smoking prevalence, the rate of quit attempts, the success rate of quit attempts, and the overall quit rate were predicted from logistic regression models that tested associations of each outcome with age. Age was modelled non-linearly using restricted cubic splines to allow for flexible associations without arbitrary categorisation. We compared models with three, four, and five knots using the Akaike information criterion (AIC) and reported the best-fitting model for each outcome (selected as the model with the lowest AIC value or the simplest model within 2 AIC units; see Additional file 1: Table S1). To examine differences by socioeconomic position, we repeated the models with the inclusion of the two-way interaction between age and social grade.

We plotted predicted modelled estimates for each year of age (16–90 years) alongside unmodelled estimates within 5-year age bands, overall and by socioeconomic position. To provide context on different life stages, we indicated on these plots the average age at which women in Great Britain give birth to their first child (31 years) [37], the age women typically go through menopause (45–55 years) [38], and the state pension age (66 years) [39]. We also reported predicted modelled estimates for selected ages across the adult lifespan and calculated prevalence ratios (with bootstrap 95% CIs, as described above) for the difference in modelled estimates between more and less advantaged women at these ages.

In an unplanned (i.e. not pre-registered) analysis, we repeated the models among men to explore whether the patterns we observed by age and socioeconomic position were unique to women.

## Results

A total of 30,699 women responded to the survey between January 2023 and February 2025. We excluded 180 women (0.6%) with missing data on smoking status or age, leaving a final sample of 30,519 women (unweighted mean [SD] age = 52.0 [18.8] years; 31.4% social grades C2DE). Except for quit attempts, which had 167 missing cases (3.7% of participants who had smoked in the past year), there were complete data on all variables. The age distribution of the sample is summarised in Additional file 2: Table S2. We also included data from 30,844 men (unweighted mean [SD] age = 52.2 [18.0] years; 31.4% social grades C2DE) for comparison (results for men are provided in Additional file 3: Tables S5–S10, Figs. S1 and S2).

### Smoking prevalence

**Fig. 1:**
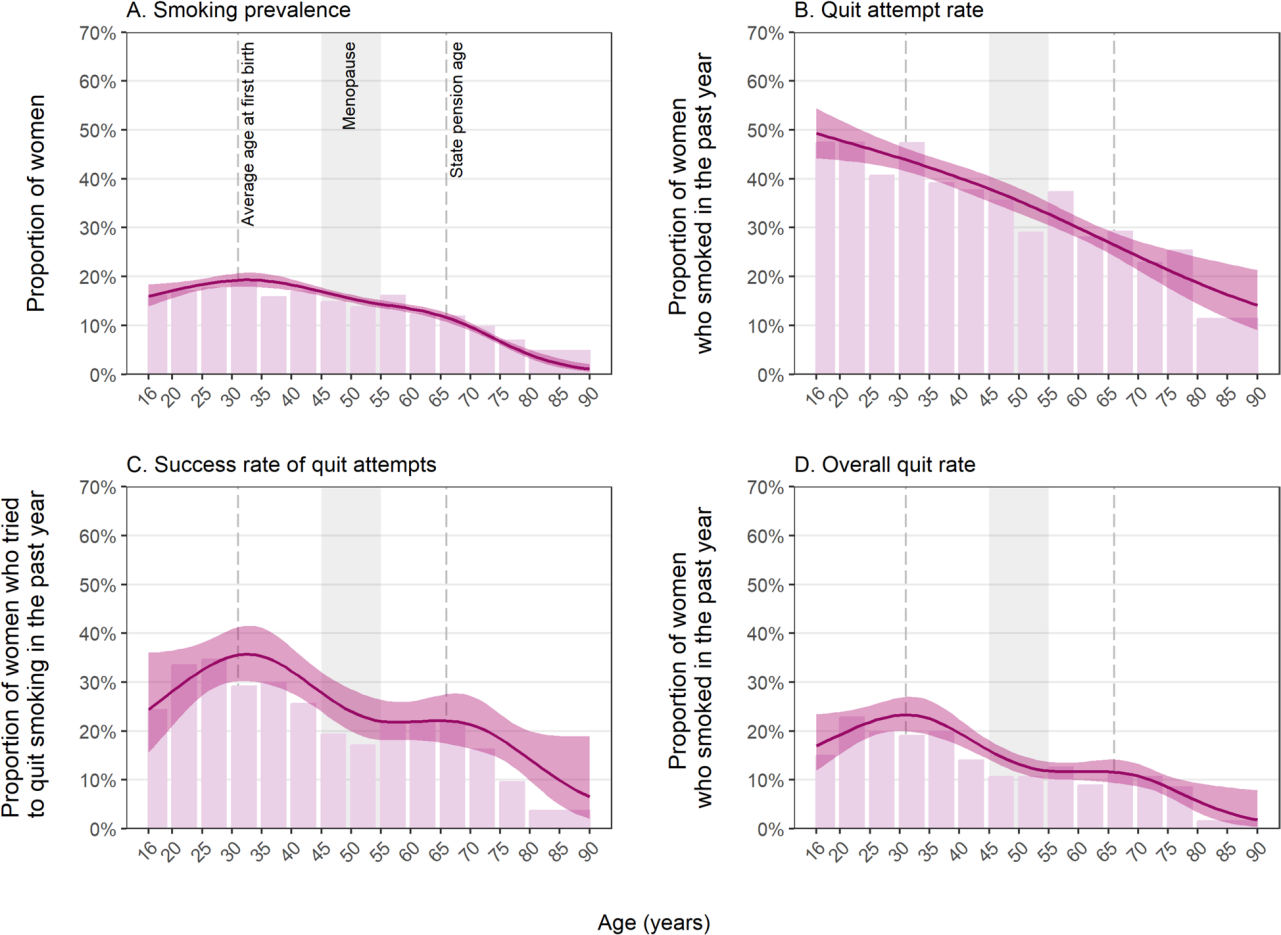
Age-specific estimates of smoking and quitting behaviours among women (≥ 16 years) in Great Britain. Lines represent modelled weighted prevalence by age, modelled non-linearly using restricted cubic splines (with five knots for current smoking, success of quit attempts, and overall quits and three knots for quit attempts; see Additional file 1: Table S1 for details of model selection). Shaded bands represent 95% confidence intervals. Bars represent unmodelled weighted prevalence estimates within age bands (also presented with 95% CIs in Additional file 2: Table S3). Unweighted sample sizes by age are provided in Additional file 2: Table S2. Corresponding figures for men are provided in Additional file 3: Fig. S1

Overall, 14.2% [13.7–14.6%] of women reported current smoking. There was a non-linear association with age (Fig. 1A). Smoking prevalence was highest among women in their 20s and 30s (peaking at 19.3% at age 33) and decreased throughout mid- and later-life (Table 1).

**Table 1:** Modelled age-specific estimates of smoking and quitting behaviours among women (≥ 16 years) in Great Britain

	% [95% CI]			
	Smoking prevalence^a^	Quit attempt rate^b^	Success rate of quit attempts^c^	Overall quit rate^b^
Age (years)^d^				
16	16.0 [13.9–18.3]	49.3 [44.2–54.4]	24.4 [15.7–36.0]	17.0 [12.0–23.5]
20	17.1 [15.6–18.8]	47.9 [43.8–52.0]	28.1 [21.1–36.5]	19.2 [15.3–23.9]
25	18.4 [17.3–19.6]	46.1 [43.1–49.2]	32.5 [27.3–38.1]	21.8 [18.8–25.2]
30	19.2 [17.9–20.5]	44.3 [41.9–46.7]	35.3 [30.1–40.9]	23.3 [20.0–26.9]
35	19.2 [17.8–20.7]	42.3 [40.1–44.6]	35.3 [29.9–41.2]	22.5 [19.2–26.3]
40	18.3 [17.2–19.5]	40.2 [37.8–42.7]	32.3 [27.8–37.1]	19.6 [17.1–22.4]
45	16.9 [16.1–17.8]	37.9 [35.4–40.6]	27.8 [24.0–32.1]	16.0 [14.2–18.1]
50	15.5 [14.7–16.3]	35.5 [32.9–38.1]	24.0 [19.9–28.6]	13.2 [11.5–15.2]
55	14.4 [13.6–15.2]	32.8 [30.5–35.2]	22.0 [18.2–26.4]	11.8 [10.2–13.7]
60	13.4 [12.6–14.3]	30.0 [27.9–32.1]	21.9 [18.2–26.1]	11.7 [10.0–13.6]
65	12.0 [11.1–13.0]	27.1 [24.8–29.4]	22.1 [17.6–27.3]	11.6 [9.5–14.2]
70	9.7 [8.9–10.6]	24.2 [21.3–27.3]	21.3 [16.4–27.2]	10.8 [8.7–13.3]
75	6.8 [6.1–7.5]	21.4 [17.7–25.5]	18.5 [14.4–23.4]	8.5 [6.6–10.8]
80	4.0 [3.2–5.0]	18.7 [14.4–24.0]	14.2 [9.8–20.0]	5.7 [3.4–9.4]

*CI* confidence interval

^a^Among women

^b^Among women who smoked in the past year

^c^Among women who tried to quit smoking in the past year

^d^Predicted weighted estimates for individual years of age from logistic regression models with age modelled using restricted cubic splines (see Additional file 1: Table S1 for details of model selection). Note that the models used to derive these estimates included data from participants of all ages

Unmodelled weighted estimates within age bands are provided in Additional file 2: Table S3. Corresponding data for men are provided in Additional file 3: Table S6

**Fig. 2:**
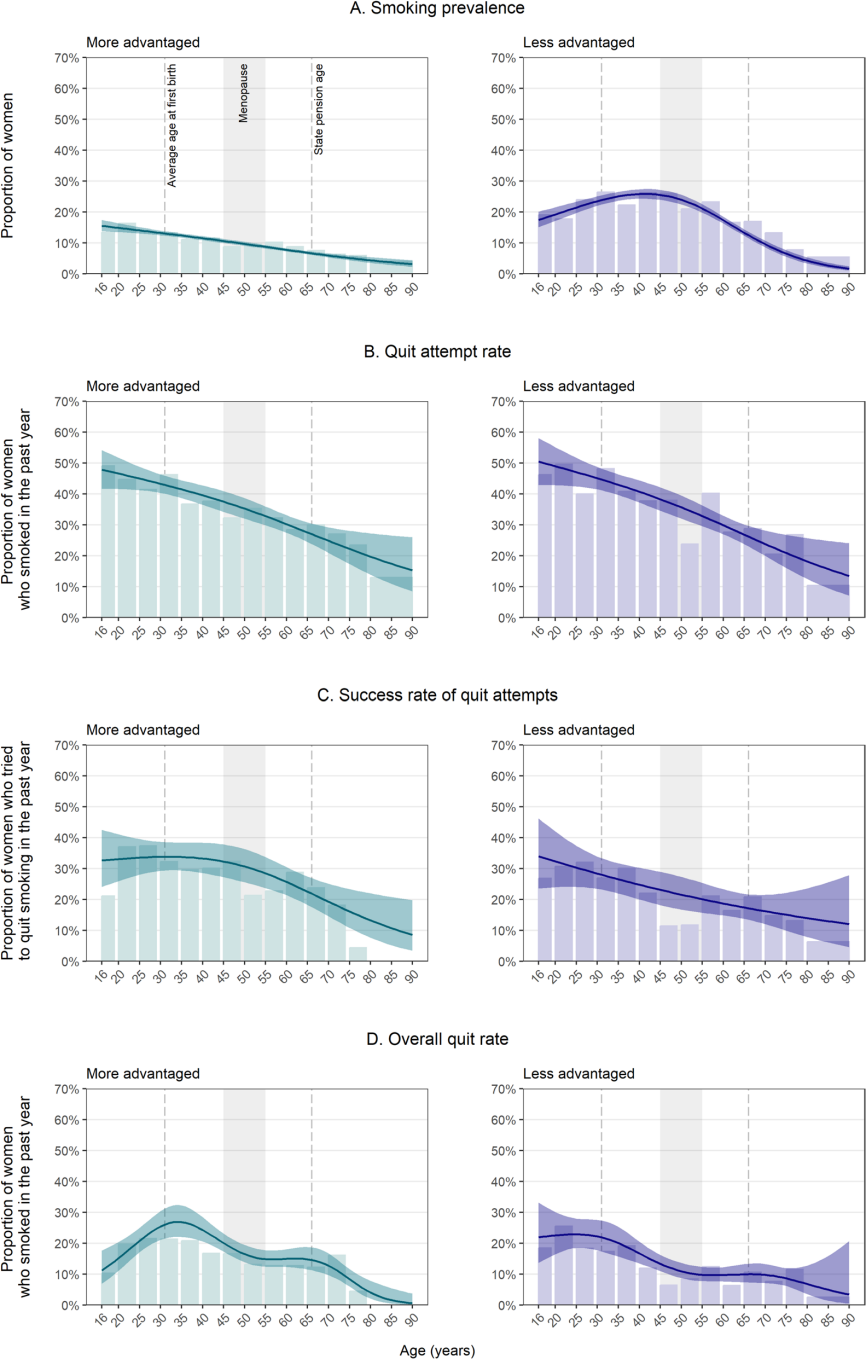
Age-specific estimates of smoking and quitting behaviours by socioeconomic position among women (≥ 16 years) in Great Britain. Lines represent modelled weighted prevalence by age modelled non-linearly using restricted cubic splines (with three knots for current smoking, quit attempts, and success of quit attempts and five knots for overall quits; see Additional file 1: Table S1 for details of model selection) and occupational social grade (ABC1 = more advantaged; C2DE = less advantaged). Shaded bands represent 95% confidence intervals. Bars represent unmodelled weighted prevalence estimates within age bands (also presented with 95% CIs in Additional file 2: Table S4). Unweighted sample sizes by age are provided in Additional file 2: Table S2. Corresponding figures for men are provided in Additional file 3: Fig. S2

There were differences in smoking prevalence by socioeconomic position. Overall, prevalence was almost twice as high among women from less compared with more advantaged social grades (19.1% [18.2–20.0%] vs. 10.4% [9.9–10.8%]; PR = 1.85 [1.72–1.99]). In addition, while smoking prevalence declined linearly with age among more advantaged women (from a high of 15.6% at age 16), there was a curvilinear association with age among those who were less advantaged (peaking at 25.9% at age 41) (Fig. 2A). These different age-related patterns meant that while smoking prevalence was similar by socioeconomic position at either end of the age spectrum, it was significantly higher among less advantaged women between the ages of 20 and 70—with a particularly high disparity in mid-life (PR ≥ 2 between ages 35 and 64; Table 2). Patterns in smoking prevalence by age and socioeconomic position were broadly similar among men (Additional file 3).

**Table 2:** Modelled age-specific estimates of smoking prevalence and the quit attempt rate by socioeconomic position among women (≥ 16 years) in Great Britain

Occupational social grade^c^	Smoking prevalence^a^			Quit attempt rate^b^		
	% [95% CI]			% [95% CI]		
	ABC1	C2DE	PR [95% CI]^d^	ABC1	C2DE	PR [95% CI]^d^
Age (years)^e^						
16	15.6 [13.8–17.5]	17.5 [15.1–20.1]	1.12 [0.92–1.34]	47.9 [41.6–54.2]	50.5 [42.8–58.1]	1.05 [0.84–1.34]
20	14.9 [13.5–16.4]	19.2 [17.1–21.5]	1.29 [1.10–1.50]	46.6 [41.6–51.7]	49.0 [42.8–55.1]	1.05 [0.88–1.28]
25	14.1 [13.1–15.1]	21.5 [19.7–23.4]	1.53 [1.36–1.70]	45.0 [41.2–48.7]	47.0 [42.5–51.7]	1.05 [0.91–1.22]
30	13.2 [12.5–14.0]	23.5 [22.0–25.1]	1.78 [1.62–1.93]	43.3 [40.3–46.3]	45.1 [41.5–48.7]	1.04 [0.94–1.18]
35	12.4 [11.8–13.1]	25.1 [23.7–26.5]	2.02 [1.85–2.20]	41.5 [38.6–44.4]	43.0 [39.6–46.4]	1.04 [0.94–1.16]
40	11.5 [10.9–12.2]	25.8 [24.3–27.4]	2.24 [2.03–2.45]	39.6 [36.5–42.7]	40.7 [37.2–44.3]	1.03 [0.92–1.18]
45	10.6 [10.0–11.3]	25.5 [24.0–27.2]	2.40 [2.17–2.65]	37.5 [34.2–40.9]	38.3 [34.6–42.1]	1.02 [0.89–1.20]
50	9.7 [9.1–10.4]	24.0 [22.5–25.6]	2.47 [2.23–2.74]	35.2 [32.0–38.6]	35.7 [32.0–39.5]	1.01 [0.87–1.20]
55	8.7 [8.2–9.3]	21.2 [19.8–22.5]	2.42 [2.18–2.69]	32.8 [29.8–35.9]	32.8 [29.5–36.3]	1.00 [0.86–1.18]
60	7.8 [7.3–8.3]	17.4 [16.3–18.5]	2.23 [2.03–2.51]	30.2 [27.4–33.1]	29.8 [26.9–32.9]	0.99 [0.85–1.15]
65	6.8 [6.3–7.4]	13.3 [12.3–14.4]	1.95 [1.75–2.26]	27.5 [24.5–30.8]	26.8 [23.7–30.1]	0.97 [0.81–1.16]
70	5.9 [5.3–6.6]	9.6 [8.6–10.7]	1.61 [1.39–2.00]	24.8 [20.9–29.2]	23.8 [19.9–28.1]	0.96 [0.72–1.25]
75	5.1 [4.4–6.0]	6.5 [5.5–7.7]	1.27 [0.99–1.74]	22.2 [17.3–28.1]	20.9 [16.0–26.7]	0.94 [0.61–1.39]
80	4.4 [3.6–5.4]	4.2 [3.4–5.3]	0.97 [0.69–1.48]	19.7 [13.9–27.2]	18.2 [12.5–25.7]	0.92 [0.50–1.61]

*CI* confidence interval, *PR* prevalence ratio

^a^Among women

^b^Among women who smoked in the past year

^c^Occupational social grades ABC1 = more advantaged, C2DE = less advantaged

^d^Prevalence ratio calculated as prevalence among less advantaged women divided by prevalence among more advantaged women, with 95% CIs calculated using bootstrapping (1000 replications)

^e^Predicted weighted estimates for individual years of age from logistic regression models with age modelled using restricted cubic splines (with three knots; see Additional file 1: Table S1 for details of model selection). Note that the models used to derive these estimates included data from participants of all ages

Unmodelled weighted estimates within age bands are provided in Additional file 2: Table S4. Corresponding data for men are provided in Additional file 3: Table S7

### Quit attempt rate

Among women who had smoked in the past year and provided data on quit attempts (*n* = 4314), 37.5% [35.9–39.2%] reported having tried to quit in the past year. There was a near-linear negative association between age and quit attempts (Fig. 1B). For example, rates of quit attempts were 47.9%, 40.2%, 30.0%, and 18.7% among 20-, 40-, 60-, and 80-year-olds who smoked (Table 1).

The prevalence of quit attempts and patterns by age were similar by socioeconomic position (Fig. 2B) and among men (Additional file 3). Overall, 37.5% [35.4–39.7%] of more advantaged women and 37.6% [35.1–40.0%] of less advantaged women reported having tried to quit in the past year (PR = 1.00 [0.92–1.10]) and the model indicated no substantial difference in prevalence at any age (with PRs ranging from 0.92 to 1.05; Table 2). However, the unmodelled data indicated a notable dip in the quit attempt rate among less advantaged women between the ages of 50 and 54 (Fig. 2B), with 23.7% of this group trying to quit in the past year compared with 38.0% and 40.2% of those aged 45–49 and 55–59, respectively (Additional file 2: Table S4).

### Success rate of quit attempts

Among women who had attempted to quit smoking in the past year (*n* = 1558), 26.2% [23.7–28.7%] were still not smoking at the time of the survey. This success rate varied non-linearly by age (Fig. 1C). Success rates were highest among women in their 20s and 30s (peaking at 35.7% at age 33), declined between the mid-30s and mid-50s, were approximately stable between the mid-50s and late 60s (ranging between 21.8 and 22.1% between ages 55 and 69), and then declined further into old age (Table 1). By contrast, the success rate of quit attempts was more similar across ages among men (Additional file 3: Fig. S1C).

Overall, the success rate of quit attempts was slightly lower among women who were less advantaged (23.8% [20.3–27.4%] vs. 29.5% [26.2–32.9%] among more advantaged women; PR = 0.81 [0.64–0.96]). This was driven by differences in quit success in mid-life: success rates were similar by socioeconomic position among women in their late teens and early 20s, but declined steadily with age among less advantaged women while remaining more stable among more advantaged women up to their 50s (Fig. 2C). As a result, the success rate of quit attempts was significantly lower among less advantaged women between the ages of 45 and 60 (with PRs ranging from 0.70 to 0.73; Table 3). The unmodelled data suggested there was a notable dip in the success rate of quit attempts among less advantaged women between the ages of 45 and 54 (Fig. 2C), with just 11.4% and 11.7% of those aged 45–49 and 50–54 who tried to stop smoking still abstinent at the time of the survey, compared with 22.0% and 21.1% of those aged 40–44 and 55–59, respectively (Additional file 2: Table S4). This was not observed among less advantaged men (Additional file 3: Fig. S2C). However, we note that sample sizes within each age group were relatively small and 95% CIs around these estimates overlapped, indicating some uncertainty in this finding.

**Table 3:** Modelled age-specific estimates of the success rate of quit attempts and overall quit rate by socioeconomic position among women (≥ 16 years) in Great Britain

Occupational social grade^c^	Success rate of quit attempts^a^			Overall quit rate^b^		
	% [95% CI]			% [95% CI]		
	ABC1	C2DE	PR [95% CI]^d^	ABC1	C2DE	PR [95% CI]^d^
Age (years)^e^						
16	32.7 [24.1–42.6]	34.0 [23.6–46.2]	1.04 [0.63–1.65]	11.2 [6.9–17.6]	21.9 [13.7–33.1]	1.96 [1.03–3.96]
20	33.1 [26.0–41.1]	32.4 [24.0–42.0]	0.98 [0.65–1.43]	15.2 [11.1–20.4]	22.5 [16.4–30.1]	1.49 [1.02–2.44]
25	33.6 [28.0–39.6]	30.3 [24.1–37.4]	0.90 [0.67–1.23]	20.8 [17.1–25.0]	22.9 [18.5–28.0]	1.10 [0.88–1.62]
30	33.8 [29.3–38.7]	28.4 [23.5–33.9]	0.84 [0.66–1.09]	25.4 [21.0–30.4]	22.2 [17.7–27.5]	0.87 [0.69–1.28]
35	33.8 [29.4–38.4]	26.6 [22.0–31.7]	0.79 [0.62–1.02]	26.9 [22.1–32.3]	20.1 [15.7–25.3]	0.75 [0.57–1.13]
40	33.3 [28.6–38.4]	24.8 [20.1–30.3]	0.75 [0.56–1.00]	24.3 [20.6–28.4]	16.7 [13.4–20.5]	0.69 [0.53–0.98]
45	32.3 [27.2–37.8]	23.2 [18.2–29.0]	0.72 [0.52–0.98]	20.0 [17.3–23.1]	13.3 [10.9–16.2]	0.67 [0.51–0.88]
50	30.7 [25.6–36.3]	21.6 [16.8–27.3]	0.70 [0.50–0.95]	16.5 [13.8–19.4]	10.9 [8.7–13.6]	0.66 [0.48–0.91]
55	28.5 [23.7–33.8]	20.1 [15.7–25.4]	0.71 [0.49–0.94]	14.9 [12.4–17.7]	9.8 [7.7–12.3]	0.66 [0.47–0.94]
60	25.7 [21.4–30.5]	18.7 [14.9–23.3]	0.73 [0.48–0.94]	14.9 [12.3–18.0]	9.7 [7.6–12.2]	0.65 [0.47–0.98]
65	22.6 [18.3–27.5]	17.4 [13.7–21.9]	0.77 [0.44–1.02]	14.8 [11.5–18.8]	9.9 [7.3–13.2]	0.67 [0.45–1.10]
70	19.3 [14.6–25.1]	16.2 [12.0–21.5]	0.84 [0.40–1.22]	12.7 [9.6–16.7]	9.7 [7.1–13.3]	0.76 [0.47–1.29]
75	16.2 [10.9–23.4]	15.1 [10.0–22.2]	0.93 [0.34–1.61]	8.3 [6.0–11.6]	8.6 [6.1–11.9]	1.03 [0.44–1.66]
80	13.3 [7.7–22.0]	14.0 [7.9–23.6]	1.06 [0.27–2.30]	4.1 [2.0–7.9]	6.8 [3.5–12.7]	1.67 [0.23–3.56]

*CI* confidence interval, *PR* prevalence ratio

^a^Among women who tried to quit smoking in the past year

^b^Among women who smoked in the past year

^c^Occupational social grades ABC1 = more advantaged, C2DE = less advantaged

^d^Prevalence ratio calculated as prevalence among less advantaged women divided by prevalence among more advantaged women, with 95% CIs calculated using bootstrapping (1000 replications)

^e^Predicted weighted estimates for individual years of age from logistic regression models with age modelled using restricted cubic splines (with three knots for the success rate of quit attempts and five knots for the overall quit rate; see Additional file 1: Table S1 for details of model selection). Note that the models used to derive these estimates included data from participants of all ages

Unmodelled weighted estimates within age bands are provided in Additional file 2: Table S4. Corresponding data for men are provided in Additional file 3: Table S8

### Overall quit rate

Among women who had smoked in the past year (*n* = 4481), 15.1% [13.8–16.3%] were not currently smoking at the time of the survey. This overall quit rate—which reflects both quit attempts and quit success—followed a similar pattern by age as quit success rates (Fig. 1D). It was highest among women in their 20s and early 30s (peaking at 23.3% at age 31), declined between the mid-30s and mid-50s, was approximately stable between the mid-50s and mid-60s (ranging between 11.6 and 11.8% between ages 55 and 66), and then declined further into old age. These age differences were more pronounced than those observed among men (Additional file 3: Fig. S1D).

Overall, there was an uncertain lower quit rate among women who were less advantaged (14.0% [12.3–15.7%] vs. 16.5% [14.9–18.2%]; PR = 0.84 [0.70–1.00]), but this was not observed consistently across ages (Fig. 2D). Among women in their late teens and early 20s, the quit rate was significantly *higher* among those who were less vs. more advantaged (e.g. 21.9% vs. 11.2% among 16-year-olds, PR = 1.96 [1.03–3.96] and 22.5% vs. 15.2% among 20-year-olds, PR = 1.49 [1.02–2.44]; Table 3), something not seen among men (Additional file 3: Table S8). However, the opposite pattern was observed between the ages of 40 and 60, where the quit rate was significantly *lower* among less advantaged women (with PRs ranging from 0.65 to 0.69; Table 3). The overall quit rate was highest at age 34 (26.9%) among more advantaged women and at age 25 (22.9%) among less advantaged women (Fig. 2D).

## Discussion

These results highlight considerable age-related and socioeconomic differences in smoking behaviours and cessation outcomes among women in Great Britain. Consistent with evidence that smoking initiation is more common in adolescence and young adulthood [40, 41], we found that smoking prevalence was highest among women in their 20s and 30s, then declined with age. However, this decline did not appear to be driven by increasing quit attempts with age. In fact, the likelihood of making a quit attempt was *lower* at older ages, suggesting that the reduction in smoking prevalence across the life course may be due to generational differences in smoking uptake and cessation [26], selective quitting by those most motivated or able to stop earlier in life, or higher mortality among older women who smoke [42, 43].

Among women who tried to quit smoking, those who were younger—especially those in their 20s and early 30s—tended to be more successful. This may be because smoking behaviours are less established at younger ages [28]. Studies show that younger people who smoke tend to have lower levels of dependence [28], and across all ages, lower dependence is a strong predictor of quit success [28, 44]. Additionally, younger women may be more influenced by social factors, viewing smoking as a way to bond with peers or as a socially normative behaviour [21]; many identify as ‘social smokers’ and restrict use to specific settings [45]. When they make the transition from this stage of their life to one that may be more routine and structured because of employment, family commitments, and other commitments, some women may find it easier to quit [21, 46, 47]. In contrast, older women, particularly those in their mid-40s to early 60s, experience less success in quitting. This difference may partly stem from self-selection: women who quit earlier in life are no longer represented in older age groups, leaving behind a cohort of older women who smoke who have longer smoking histories and higher dependence [28]. It is also possible that women experiencing perimenopausal and menopausal symptoms [48] may be less likely to initiate a quit attempt and may find it more difficult to quit [49]. There could be biological explanations for this, and/or social reasons such as a greater number of life and work commitments which mean quitting is not prioritised.

Notably, age patterns differed by socioeconomic position. While smoking declined steadily with age among more advantaged women, it followed a curvilinear pattern among less advantaged women, peaking in the early 40s. As a result, smoking prevalence was more than twice as high among less advantaged than more advantaged women between the ages of 35 and 64, highlighting mid-life as a period of particularly marked inequality. A similar pattern was observed among men. These disparities may reflect the cumulative burden of greater life stresses, more physically demanding work and caregiving roles (including, for many, the ‘double burden’ of caring for still dependent children and also ageing parents), greater tacit approval toward smoking within their social network, and limited access to tailored cessation support—factors that disproportionately affect people with fewer resources [50]. For many, living within an environment where smoking is culturally and socially acceptable and even an expected coping strategy means that quit attempts are harder to access and undermined, particularly in the absence of viable alternatives to deal with stress [30, 51]. As smoking as a stress response is a learned behaviour [52], the association of the act of smoking with a perceived reduction in stress will likely become stronger and more embedded as it is continually reinforced with ageing. Moreover, older women from less advantaged backgrounds may also place a low priority on their own health, which can lead them to continue smoking even in the face of serious health problems [51]. These combined material, social, and cultural factors can make smoking more deeply embedded in daily life, intensifying challenges to quitting and perpetuating health and care inequalities. Taking a broad social approach to helping these women quit is a particular challenge moving forward for cessation providers. Further, occupational factors such as job security, working conditions, and workplace stress may also contribute to these socioeconomic inequalities in smoking behaviours and cessation outcomes, highlighting the need for policies that address structural and occupational determinants of health.

Despite these structural barriers, quit attempt rates were broadly similar across socioeconomic groups, suggesting less advantaged women were just as motivated to quit. This echoes previous research documenting high levels of motivation to stop smoking in disadvantaged population groups [53, 54]. However, there was some evidence of a dip in quit attempts among less advantaged women between the ages of 50 and 54, a pattern not seen among their more advantaged peers or among less advantaged men of the same age. This may reflect the impact of mid-life transitions such as perimenopause and menopause, which are often accompanied by mood changes, increased stress, and other health challenges [48]. Hormonal changes during perimenopause and menopause can influence nicotine metabolism and withdrawal experiences, potentially increasing dependence and reducing cessation success [49]. Previous studies have suggested that women from less advantaged backgrounds experience worse menopausal symptoms [55–57], potentially increasing their reliance on smoking as a coping mechanism. At the same time, women in this age group are often balancing multiple responsibilities—caring for ageing parents, supporting adult children and grandchildren, and maintaining paid work [58]—which may leave little time or energy to prioritise their own health needs, including quitting. For those from disadvantaged backgrounds, these pressures are compounded by intersectional risk factors, creating overlapping levels of disadvantage, such as reduced access to healthcare, financial insecurity, and lack of social support [58].

Furthermore, we found that even when quit attempts were made, success rates were consistently lower among less advantaged women, particularly in mid-life (ages 45 to 60). This aligns with previous findings that people from less advantaged backgrounds experience greater difficulty in quitting smoking [30, 53, 59, 60], likely due to earlier smoking initiation, stronger dependence, and fewer social and cultural resources to support quitting [30]. The overall quit rate was highest at age 31 for more advantaged women and at age 25 for less advantaged women. This broadly maps onto differences in the average age of childbirth by socioeconomic position [61] and may reflect smoking cessation during pregnancy—a strong motivator for quitting [21, 22]. More advantaged women, who tend to have children later [61] and often have greater access to resources and support [30], tend to be more likely to maintain abstinence post-partum [62]. These patterns may help explain the sustained decline in smoking prevalence observed among more advantaged women earlier in life.

Understanding barriers to cessation faced by women at different life stages and from different socioeconomic backgrounds is important for developing more effective, equitable, and targeted cessation interventions. Tailoring programmes to address the specific challenges faced by older and less advantaged women—such as support with managing stress, hormonal changes, or caregiving responsibilities—could be key to improving cessation outcomes in these groups. For example, stress relief is often cited as a primary reason for smoking [63, 64], particularly among women [63], who may view cigarettes as a vital coping mechanism. However, this belief is at odds with research showing that perceived stress levels actually *decrease* after quitting smoking [52, 65, 66]. Highlighting this evidence could help reshape these perceptions and reduce psychological resistance to quitting. Furthermore, acknowledging the social and familial demands placed on mid-life women could help make cessation support feel more responsive, supportive, and realistic, which may improve outcomes across the life course. Finally, targeted smoking cessation interventions for women who continue to smoke entering the menopausal transition may be a fruitful area for intervention development. Further, qualitative research with women who smoke of different ages and socioeconomic backgrounds would be important to inform the development of any such interventions.

From an international perspective, these findings also have broader implications for tobacco control and public health policy. The socioeconomic and gendered patterns observed among women in Great Britain are consistent with evidence from other high-income countries showing that smoking is increasingly concentrated among people with fewer resources and that women face unique social and biological barriers to quitting [30, 67, 68]. To reduce these inequities, international tobacco control strategies could benefit from adopting a gender-responsive approach—for example, by integrating cessation support into reproductive and primary care services, ensuring that national quit programmes are accessible and relevant to women of different ages and socioeconomic backgrounds, and addressing structural determinants such as childcare responsibilities and financial stress. The WHO Framework Convention on Tobacco Control (FCTC) emphasises the need to consider gender and social inequalities in tobacco policy [69], and our findings highlight the importance of strengthening these commitments through equitable access to cessation interventions globally [70].

This study has several limitations. First, the cross-sectional nature of the data limits our ability to draw causal conclusions about the relationships between age, socioeconomic position, and smoking behaviours. It is not clear how far the age-related differences observed reflect cohort effects as opposed to changes within individuals across the life course. Longitudinal studies would help better understand how smoking behaviours and cessation outcomes evolve over time, particularly during life stage transitions such as pregnancy, menopause, and retirement. Second, the use of self-reported data may introduce bias, as people may underreport smoking or misremember quit attempts, particularly among those who quit some time ago. Third, we used a broad binary measure of occupational social grade; future research using finer distinctions or alternative indicators of socioeconomic position (e.g. education or income) may provide additional insight into the mechanisms underpinning these inequalities. Finally, despite the large overall sample size, there were relatively small numbers of participants within certain age bands, particularly in the older age ranges and when restricting the sample to those who had smoked or attempted to quit in the past year. This could affect the precision of some estimates and contribute to uncertainty in the findings. However, the use of restricted cubic splines to model age-related differences helps mitigate this issue by incorporating data from participants across all ages to estimate the prevalence of a given outcome for each specific year of age. This approach provides greater statistical power than treating age as a categorical variable, allowing for more accurate and nuanced age-specific estimates.

## Conclusions

This study underscores the importance of considering both age and socioeconomic position when designing smoking cessation interventions for women. Tailored strategies that respond to the unique challenges and life circumstances of women at different ages—especially those in mid-life and from less advantaged backgrounds—could improve smoking cessation outcomes and reduce health inequalities. Future research should explore the underlying factors that contribute to these age-related and socioeconomic differences in smoking behaviours and cessation success, to inform the development of targeted interventions.

## Supplementary Information

Additional file 1. Model selection. Table S1 Model selection: AIC values for models with 3, 4, and 5 knots.

Additional file 2. Additional results for women. Table S2 Distribution of participants by age. Table S3 Observed age-specific estimates of smoking and quitting behaviours among women (≥ 16 years) in Great Britain. Table S4 Observed age-specific estimates of smoking and quitting behaviours by socioeconomic position among women (≥ 16 years) in Great Britain.

Additional file 3. Results for men. Table S5 Distribution of participants by age. Fig. S1 Age-specific estimates of smoking and quitting behaviours among men (≥ 16 years) in Great Britain. Table S6 Modelled age-specific estimates of smoking and quitting behaviours among men (≥ 16 years) in Great Britain. Fig. S2 Age-specific estimates of smoking and quitting behaviours by socioeconomic position among men (≥ 16 years) in Great Britain. Table S7 Modelled age-specific estimates of smoking prevalence and the quit attempt rate by socioeconomic position among men (≥ 16 years) in Great Britain. Table S8 Modelled age-specific estimates of the success rate of quit attempts and overall quit rate by socioeconomic position among men (≥ 16 years) in Great Britain. Table S9 Observed age-specific estimates of smoking and quitting behaviours among men (≥ 16 years) in Great Britain. Table S10 Observed age-specific estimates of smoking and quitting behaviours by socioeconomic position among men (≥ 16 years) in Great Britain.

## Data Availability

Data used in these analyses are available on Open Science Framework ( https:/osf.io/w2smk ), with age provided in bands to preserve anonymity.

## Abbreviations

ABC1: More advantaged occupational social grades
AIC: Akaike information criterion
C2DE: Less advantaged occupational social grades
CI: Confidence interval
PR: Prevalence ratio
