# Supplementary material for: Patterns of disparity: age and socioeconomic differences in women’s smoking and quitting outcomes in Great Britain
Source: BMC Med. 2026 Feb 10;24:16. doi: 10.1186/s12916-025-04574-1 (PMC12888745; doi:10.1186/s12916-025-04574-1)
Supplement: Supplementary file 1 — Additional file 1. Model selection. Table S1 Model selection: AIC values for models with 3, 4, and 5 knots. [file 12916_2025_4574_MOESM1_ESM.pdf]

Additional file 1: model selection

Table S1. Model selection: AIC values for models with 3, 4, and 5 knots

	AIC		
	**3 knots**	**4 knots**	**5 knots**
Smoking prevalence			
All women	24524.74	24523.26	24518.06
Women by social grade	23926.80	23927.89	23928.11
All men	27934.53	27936.25	27930.41
Men by social grade	27364.54	27363.77	27359.70
Quit attempt rate			
All women	5632.81	5635.01	5635.31
Women by social grade	5638.94	5643.44	5645.38
All men	6531.202	6530.439	6531.541
Men by social grade	6528.703	6528.898	6528.730
Success rate of quit attempts			
All women	1773.56	1774.28	1771.10
Women by social grade	1772.74	1775.20	1773.71
All men	2062.517	2064.162	2066.865
Men by social grade	2046.305	2044.683	2047.948
Overall quit rate			
All women	3733.97	3734.27	3727.19
Women by social grade	3724.74	3726.77	3719.46
All men	4139.289	4136.484	4136.637
Men by social grade	4119.747	4120.408	4123.208

AIC, Akaike Information Criterion.
Shaded cells indicate the best fitting model (the model with the lowest AIC or the simplest model within 2 AIC units).
