# Supplementary material for: Patterns of disparity: age and socioeconomic differences in women’s smoking and quitting outcomes in Great Britain
Source: BMC Med. 2026 Feb 10;24:16. doi: 10.1186/s12916-025-04574-1 (PMC12888745; doi:10.1186/s12916-025-04574-1)
Supplement: Supplementary file 2 — Additional file 2. Additional results for women. Table S2 Distribution of participants by age. Table S3 Observed age-specific estimates of smoking and quitting behaviours among women (≥ 16 years) in Great Britain. Table S4 Observed age-specific estimates of smoking and quitting behaviours by socioeconomic position among women (≥ 16 years) in Great Britain. [file 12916_2025_4574_MOESM2_ESM.pdf]

Additional file 2: additional results for women

Table S2. Distribution of participants by age

	**Women**			**Women from more advantaged social grades (ABC1)**			**Women from less advantaged social grades (C2DE)**		
	Total *n*	Smoked in past year *n*	Tried to quit smoking in past year *n*	Total *n*	Smoked in past year *n*	Tried to quit smoking in past year *n*	Total *n*	Smoked in past year *n*	Tried to quit smoking in past year *n*
All ages, *n*	30,519	4481	1558	20,582	2418	848	9937	2063	710
Age in years, % (*n*)									
16-19	3.4 (778)	3.8 (129)	5.0 (62)	3.3 (531)	3.9 (77)	5.3 (36)	3.6 (247)	3.8 (52)	4.8 (26)
20-24	8.8 (1938)	11.6 (413)	14.9 (192)	7.7 (1269)	12.6 (251)	15.1 (113)	10.2 (669)	10.9 (162)	14.8 (79)
25-29	6.3 (1617)	8.3 (326)	9.0 (132)	6.7 (1178)	9.4 (199)	10.3 (82)	5.7 (439)	7.5 (127)	8.0 (50)
30-34	9.9 (2448)	13.7 (508)	17.3 (225)	10.1 (1720)	13.7 (286)	17 (125)	9.5 (728)	13.7 (222)	17.6 (100)
35-39	6.5 (1671)	7.7 (309)	7.9 (117)	6.7 (1181)	7.6 (168)	7.4 (61)	6.2 (490)	7.7 (141)	8.3 (56)
40-44	9.1 (2401)	10.8 (430)	10.9 (154)	10.1 (1775)	11.2 (249)	11.3 (89)	7.8 (626)	10.5 (181)	10.6 (65)
45-49	6.5 (1926)	6.4 (295)	6.2 (106)	7.5 (1446)	6.4 (160)	5.6 (54)	5.2 (480)	6.5 (135)	6.6 (52)
50-54	9.7 (2856)	8.9 (423)	6.9 (118)	10.9 (2089)	9.7 (237)	9.2 (76)	8.1 (767)	8.3 (186)	5.3 (42)
55-59	7.4 (2493)	8.1 (417)	7.9 (143)	7.2 (1665)	6.8 (197)	5.9 (59)	7.5 (828)	9.0 (220)	9.4 (84)
60-64	8.4 (2878)	6.8 (368)	5.0 (96)	8.1 (1921)	6.5 (185)	5.0 (49)	8.7 (957)	7.0 (183)	5.1 (47)
65-69	6.9 (2582)	5.5 (323)	4.3 (98)	6.6 (1690)	4.6 (148)	3.5 (43)	7.3 (892)	6.2 (175)	4.8 (55)
70-74	6.4 (2468)	4.2 (260)	2.5 (65)	5.8 (1531)	3.5 (116)	2.5 (35)	7.2 (937)	4.7 (144)	2.4 (30)
75-79	6.1 (2433)	2.7 (178)	1.7 (37)	5.5 (1478)	2.7 (98)	1.7 (20)	6.9 (955)	2.7 (80)	1.8 (17)
≥80	4.8 (2030)	1.4 (102)	0.4 (13)	3.8 (1108)	1.2 (47)	0.4 (6)	6.1 (922)	1.5 (55)	0.4 (7)

Data are shown as weighted percentages and unweighted sample sizes.

Additional file 2: additional results for women

Table S3. Observed age-specific estimates of smoking and quitting behaviours among women (≥16y) in Great Britain

	% [95% CI]			
	**Smoking prevalence**[1]	**Quit attempt rate**[2]	**Success rate of quit attempts**[3]	**Overall quit rate**[2]
Age (years)[4]				
16-19	16.1 [13.0–19.1]	47.4 [37.7–57.1]	24.3 [12.2–36.4]	14.9 [7.5–22.3]
20-24	17.0 [15.1–18.9]	47.4 [41.9–52.9]	33.5 [25.9–41.0]	22.8 [18.2–27.4]
25-29	17.7 [15.5–19.8]	40.6 [34.6–46.7]	34.6 [25.6–43.6]	19.8 [15.1–24.5]
30-34	18.8 [17.0–20.6]	47.3 [42.3–52.3]	29.1 [22.6–35.7]	19.0 [15.2–22.7]
35-39	15.8 [13.7–17.8]	39.1 [32.8–45.4]	29.9 [20.5–39.3]	19.8 [14.9–24.7]
40-44	17.0 [15.2–18.7]	37.7 [32.4–42.9]	25.6 [18.0–33.1]	14.0 [10.4–17.6]
45-49	14.8 [12.9–16.7]	35.6 [29.3–41.8]	19.3 [11.6–27.0]	10.6 [7.1–14.2]
50-54	13.7 [12.3–15.2]	29.0 [24.1–33.9]	17.1 [9.9–24.3]	10.6 [7.4–13.7]
55-59	16.0 [14.3–17.8]	37.3 [31.8–42.8]	21.6 [14.1–29.2]	12.5 [9.0–16.0]
60-64	12.3 [10.9–13.7]	28.0 [22.6–33.4]	21.6 [12.8–30.4]	8.9 [5.8–11.9]
65-69	11.8 [10.4–13.3]	29.1 [23.6–34.7]	21.8 [12.9–30.8]	11.1 [7.5–14.8]
70-74	9.7 [8.4–11.1]	22.8 [17.1–28.6]	16.2 [6.5–25.9]	10.6 [6.3–14.9]
75-79	6.9 [5.6–8.1]	25.3 [16.8–33.8]	9.6 [0.4–18.7]	8.5 [3.7–13.3]
≥80	4.8 [3.8–5.8]	11.3 [4.8–17.8]	3.6 [0.0–10.8]	1.5 [0.0–3.4]

CI, confidence interval.

[1] Among women.

[2] Among women who smoked in the past year.

[3] Among women who tried to quit smoking in the past year.

[4] Observed weighted estimates within age bands. Sample sizes are provided in **Table S2**; note small denominators for some estimates (e.g., quitting outcomes in older age bands).

Additional file 2: additional results for women

Table S4. Observed age-specific estimates of smoking and quitting behaviours by socioeconomic position among women (≥16y) in Great Britain

Occupational social grade[4]	Smoking prevalence[1]		Quit attempt rate[2]		Success rate of quit attempts[3]		Overall quit rate[2]	
	**% [95% CI]**							
	**ABC1**	**C2DE**	**ABC1**	**C2DE**	**ABC1**	**C2DE**	**ABC1**	**C2DE**
Age (years)[5]								
16-19	13.5 [10.4–16.6]	19.1 [13.7–24.6]	49.0 [37.2–60.9]	46.2 [31.7–60.7]	21.2 [7.2–35.2]	26.8 [8.2–45.4]	10.3 [3.3–17.3]	18.5 [6.7–30.2]
20-24	16.3 [14.1–18.5]	17.7 [14.6–20.8]	44.7 [38.0–51.4]	49.6 [41.3–57.9]	37.1 [27.4–46.9]	30.8 [19.8–41.7]	19.6 [14.3–24.9]	25.4 [18.2–32.7]
25-29	13.6 [11.5–15.6]	24.0 [19.7–28.4]	41.4 [34.0–48.8]	40.0 [30.7–49.3]	37.3 [26.2–48.5]	32.0 [18.1–46.0]	21.5 [15.4–27.5]	18.3 [11.3–25.3]
30-34	13.3 [11.6–15.0]	26.5 [22.9–30.0]	46.2 [39.9–52.4]	48.1 [40.8–55.4]	32.2 [23.6–40.9]	26.9 [17.6–36.3]	21.3 [16.2–26.3]	17.3 [12.0–22.7]
35-39	11.2 [9.3–13.1]	22.2 [18.2–26.3]	36.7 [28.9–44.6]	40.8 [31.6–50.0]	29.7 [17.6–41.7]	30.1 [16.7–43.5]	20.8 [14.3–27.3]	19.1 [12.1–26.2]
40-44	11.5 [9.9–13.1]	26.3 [22.5–30.0]	37.6 [31.1–44.2]	37.7 [29.9–45.6]	30.2 [20.2–40.2]	22.0 [11.1–32.9]	16.6 [11.7–21.6]	11.9 [6.9–17.0]
45-49	8.8 [7.3–10.4]	26.1 [21.7–30.5]	32.2 [24.4–40.1]	38.0 [28.9–47.1]	32.3 [18.8–45.8]	11.4 [3.0–19.8]	16.6 [10.4–22.8]	6.4 [2.3–10.4]
50-54	9.6 [8.3–11.0]	21.0 [17.8–24.1]	35.3 [28.6–42.0]	23.7 [16.8–30.7]	21.3 [11.2–31.4]	11.7 [2.0–21.5]	13.1 [8.4–17.8]	8.4 [4.2–12.6]
55-59	10.2 [8.6–11.8]	23.3 [20.0–26.6]	32.3 [25.0–39.5]	40.2 [32.6–47.7]	22.7 [11.3–34.2]	21.1 [11.4–30.9]	12.8 [7.7–17.8]	12.4 [7.6–17.1]
60-64	8.7 [7.3–10.1]	16.6 [14.0–19.3]	28.5 [21.3–35.6]	27.7 [20.0–35.3]	28.8 [15.1–42.5]	16.5 [5.3–27.7]	12.7 [7.5–17.8]	6.2 [2.7–9.8]
65-69	7.5 [6.1–8.9]	17.0 [14.2–19.7]	30.0 [21.6–38.3]	28.7 [21.5–35.9]	23.8 [10.6–36.9]	20.8 [9.0–32.6]	12.2 [6.9–17.4]	10.5 [5.7–15.4]
70-74	6.3 [5.0–7.7]	13.3 [10.9–15.7]	27.0 [18.2–35.8]	20.5 [13.1–27.9]	18.2 [3.0–33.3]	14.8 [2.3–27.3]	16.1 [8.4–23.8]	7.6 [2.5–12.7]
75-79	6.0 [4.7–7.3]	7.8 [5.7–9.8]	23.4 [13.6–33.2]	26.8 [13.7–39.8]	4.4 [0.0–10.6]	13.2 [0.0–28.3]	4.4 [0.6–8.2]	11.5 [3.6–19.4]
≥80	4.1 [2.8–5.3]	5.4 [3.8–7.0]	12.9 [2.2–23.7]	10.3 [2.3–18.4]	0.0 [0.0–0.0]	6.4 [0.0–19.1]	0.0 [0.0–0.0]	2.5 [0.0–5.5]

CI, confidence interval.

[1] Among women.

[2] Among women who smoked in the past year.

[3] Among women who tried to quit smoking in the past year.

[4] Occupational social grades ABC1 = more advantaged, C2DE = less advantaged.

[5] Observed weighted estimates within age bands. Sample sizes are provided in **Table S2**; note small denominators for some estimates (e.g., quitting outcomes in older age bands).
