# Supplementary material for: Patterns of disparity: age and socioeconomic differences in women’s smoking and quitting outcomes in Great Britain
Source: BMC Med. 2026 Feb 10;24:16. doi: 10.1186/s12916-025-04574-1 (PMC12888745; doi:10.1186/s12916-025-04574-1)
Supplement: Supplementary file 3 — Additional file 3. Results for men. Table S5 Distribution of participants by age. Fig. S1 Age-specific estimates of smoking and quitting behaviours among men (≥ 16 years) in Great Britain. Table S6 Modelled age-specific estimates of smoking and quitting behaviours among men (≥ 16 years) in Great Britain. Fig. S2 Age-specific estimates of smoking and quitting behaviours by socioeconomic position among men (≥ 16 years) in Great Britain. Table S7 Modelled age-specific estimates of smoking prevalence and the quit attempt rate by socioeconomic position among men (≥ 16 years) in Great Britain. Table S8 Modelled age-specific estimates of the success rate of quit attempts and overall quit rate by socioeconomic position among men (≥ 16 years) in Great Britain. Table S9 Observed age-specific estimates of smoking and quitting behaviours among men (≥ 16 years) in Great Britain. Table S10 Observed age-specific estimates of smoking and quitting behaviours by socioeconomic position among men (≥ 16 years) in Great Britain. [file 12916_2025_4574_MOESM3_ESM.pdf]

Additional file 3: results for men

Table S5. Distribution of participants by age

	**Men**			**Men from more advantaged social grades (ABC1)**			**Men from less advantaged social grades (C2DE)**		
	Total *n*	Smoked in past year *n*	Tried to quit smoking in past year *n*	Total *n*	Smoked in past year *n*	Tried to quit smoking in past year *n*	Total *n*	Smoked in past year *n*	Tried to quit smoking in past year *n*
All ages, *n*	30,844	5299	1761	21,165	3011	1000	9679	2288	761
Age in years, % (*n*)									
16-19	4.5 (1091)	5.3 (241)	7.9 (120)	4.5 (770)	6.0 (156)	7.7 (71)	4.6 (321)	4.7 (85)	8.0 (49)
20-24	8.7 (2066)	12.3 (564)	15.9 (253)	7.6 (1365)	13.3 (358)	16.8 (160)	9.9 (701)	11.5 (206)	15.3 (93)
25-29	7.3 (1644)	10.4 (447)	12.9 (182)	7.2 (1161)	11.0 (281)	12.8 (112)	7.4 (483)	10.0 (166)	13.0 (70)
30-34	9.8 (2201)	14.9 (629)	16.3 (243)	10.2 (1593)	16.0 (387)	17.4 (150)	9.2 (608)	14.1 (242)	15.5 (93)
35-39	7.1 (1770)	8.4 (396)	8.7 (147)	7.3 (1250)	7.8 (215)	8.7 (82)	6.9 (520)	8.8 (181)	8.7 (65)
40-44	8.8 (2275)	9.2 (445)	8.0 (139)	9.7 (1680)	9.6 (262)	9.9 (91)	7.7 (595)	8.9 (183)	6.7 (48)
45-49	6.3 (1834)	6.3 (345)	5.0 (101)	6.8 (1328)	5.8 (182)	5.2 (56)	5.6 (506)	6.7 (163)	4.9 (45)
50-54	10.0 (2838)	9.5 (494)	8.2 (148)	10.3 (2001)	9.3 (273)	8.1 (82)	9.6 (837)	9.6 (221)	8.3 (66)
55-59	7.5 (2711)	7.1 (471)	6.1 (141)	7.3 (1814)	6.0 (231)	4.6 (64)	7.7 (897)	7.9 (240)	7.1 (77)
60-64	8.3 (3023)	6.2 (417)	4.2 (108)	7.7 (1995)	5.3 (213)	3.2 (48)	8.9 (1028)	6.8 (204)	4.9 (60)
65-69	6.4 (2709)	4.2 (330)	3.2 (85)	6.3 (1808)	3.7 (168)	2.6 (40)	6.6 (901)	4.6 (162)	3.6 (45)
70-74	5.9 (2509)	3.0 (241)	1.7 (41)	5.9 (1679)	2.7 (127)	1.2 (17)	6.0 (830)	3.2 (114)	2.1 (24)
75-79	5.7 (2456)	2.1 (177)	1.4 (40)	5.5 (1615)	2.1 (97)	1.3 (20)	5.9 (841)	2.2 (80)	1.5 (20)
≥80	3.8 (1717)	1.2 (102)	0.4 (13)	3.7 (1106)	1.3 (61)	0.4 (7)	3.9 (611)	1.1 (41)	0.4 (6)

Data are shown as weighted percentages and unweighted sample sizes.

Additional file 3: results for men

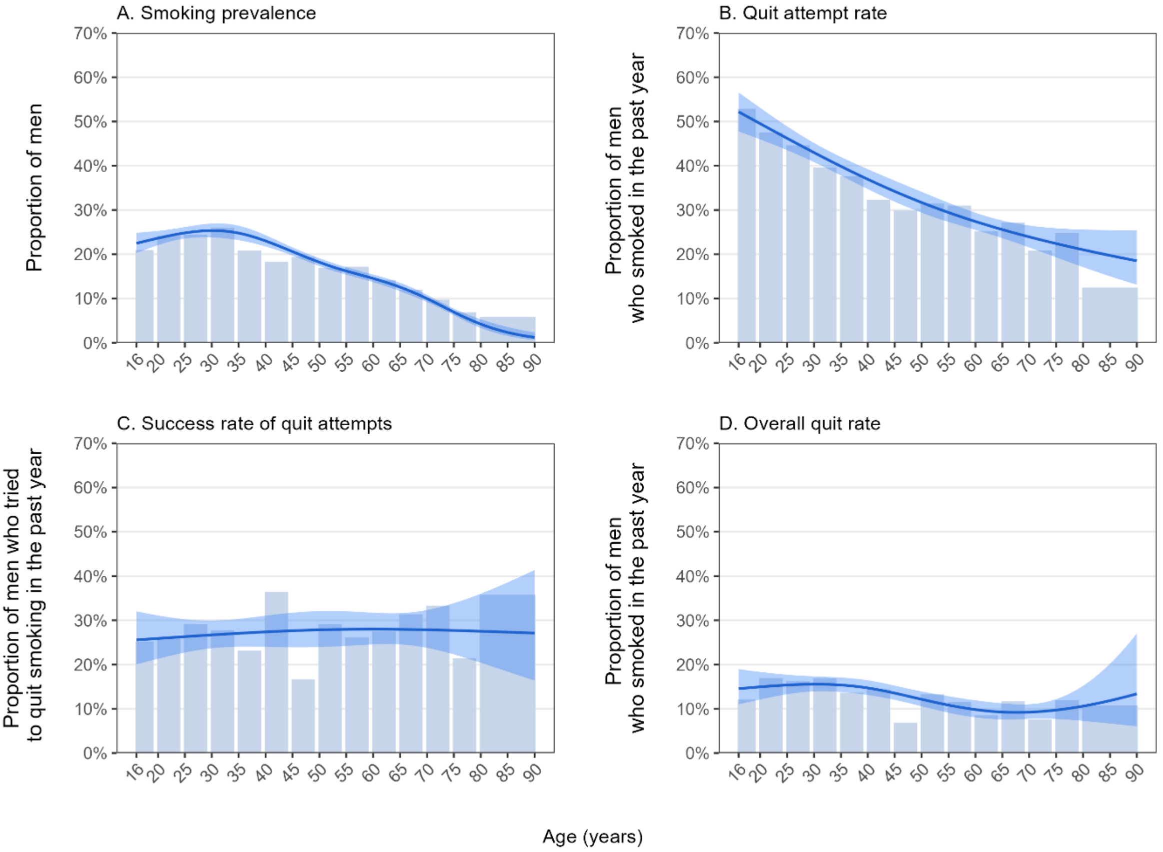

Figure S1. Age-specific estimates of smoking and quitting behaviours among men (≥16y) in Great Britain. Lines represent modelled weighted prevalence by age, modelled non-linearly using restricted cubic splines (see **Supplementary File 1** for details of model selection). Shaded bands represent 95% confidence intervals. Bars represent unmodelled weighted prevalence estimates within age bands (also presented with 95% CIs in **Table S9**). Unweighted sample sizes by age are provided in **Table S5**.

Additional file 3: results for men

Table S6. Modelled age-specific estimates of smoking and quitting behaviours among men (≥16y) in Great Britain

	% [95% CI]			
	**Smoking prevalence[1]**	**Quit attempt rate[2]**	**Success rate of quit attempts[3]**	**Overall quit rate[2]**
Age (years)[4]				
16	22.5 [20.3–24.8]	52.2 [47.8–56.6]	25.6 [20.0–32.0]	14.5 [11.0–19.0]
20	23.6 [22.1–25.3]	49.5 [46.0–53.1]	25.9 [21.3–31.1]	15.0 [12.1–18.3]
25	24.8 [23.6–26.1]	46.2 [43.5–48.9]	26.3 [22.7–30.2]	15.4 [13.3–17.7]
30	25.3 [23.8–26.9]	43.0 [40.8–45.1]	26.7 [23.7–29.9]	15.6 [13.9–17.3]
35	24.8 [23.2–26.4]	39.9 [37.8–42.0]	27.1 [24.0–30.3]	15.4 [13.8–17.1]
40	23.0 [21.8–24.3]	36.9 [34.7–39.2]	27.4 [24.0–31.0]	14.7 [13.1–16.4]
45	20.6 [19.7–21.6]	34.2 [31.9–36.6]	27.6 [23.9–31.7]	13.5 [12.0–15.2]
50	18.2 [17.3–19.1]	31.7 [29.4–34.1]	27.8 [24.0–32.1]	12.1 [10.6–13.9]
55	16.2 [15.4–17.1]	29.4 [27.3–31.6]	28.0 [24.2–32.0]	10.8 [9.1–12.8]
60	14.5 [13.7–15.4]	27.4 [25.5–29.4]	28.0 [24.5–31.7]	9.8 [8.1–11.9]
65	12.6 [11.6–13.6]	25.6 [23.6–27.6]	28.0 [24.6–31.6]	9.3 [7.6–11.2]
70	10.0 [9.2–10.8]	23.9 [21.5–26.5]	27.9 [23.8–32.3]	9.2 [7.7–11.0]
75	6.9 [6.2–7.7]	22.4 [19.3–25.9]	27.7 [22.3–33.9]	9.7 [7.7–12.2]
80	4.2 [3.3–5.3]	21.0 [17.1–25.6]	27.5 [20.4–36.0]	10.6 [7.3–15.2]

CI, confidence interval.

[1] Among men.

[2] Among men who smoked in the past year.

[3] Among men who tried to quit smoking in the past year.

[4] Predicted weighted estimates for individual years of age from logistic regression models with age modelled using restricted cubic splines (see **Supplementary File 1** for details of model selection). Note that the models used to derive these estimates included data from participants of all ages. Unmodelled weighted estimates within age bands are provided in **Table S9**.

Additional file 3: results for men

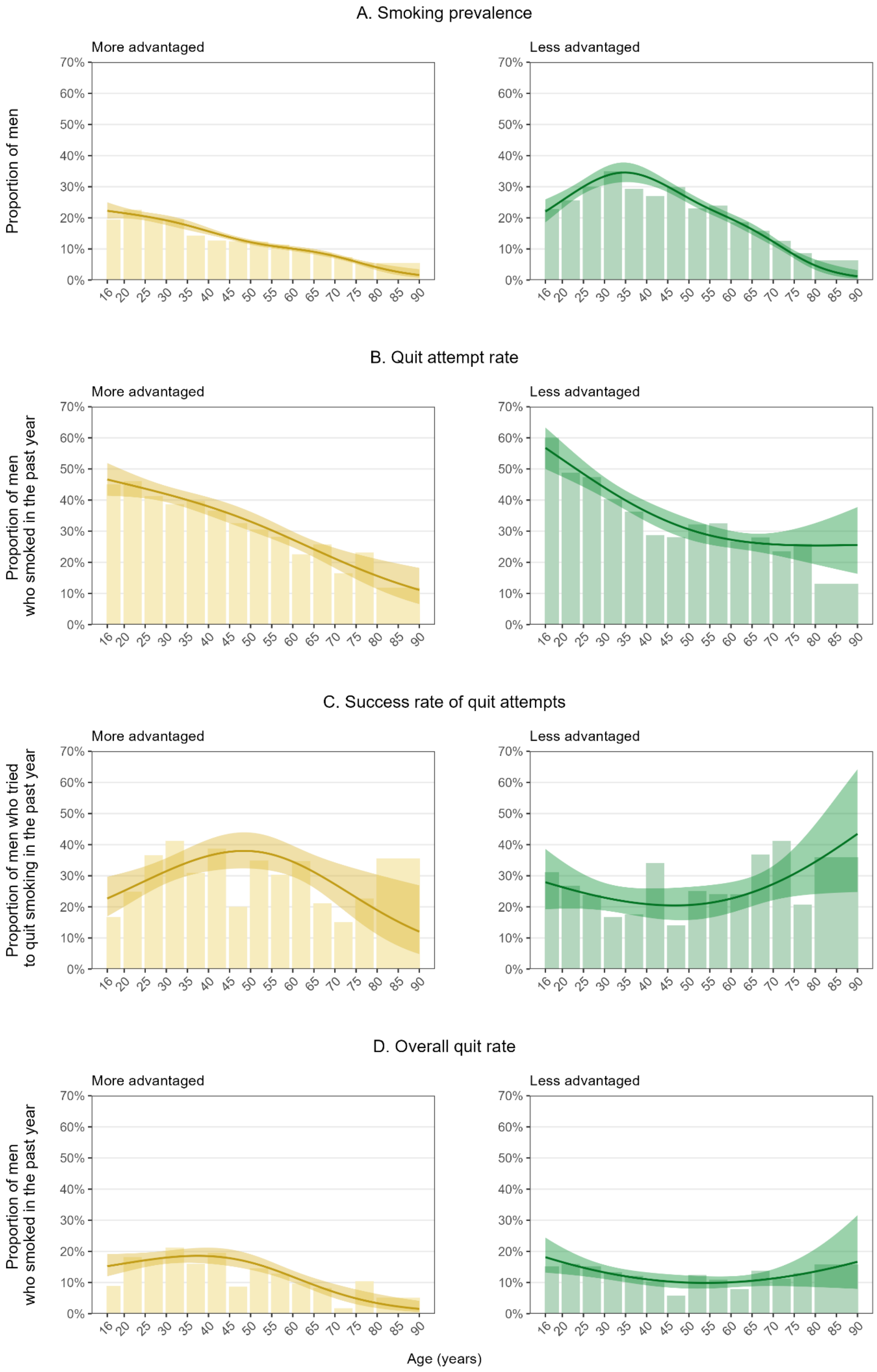

Additional file 3: results for men

Figure legend on next page.

Additional file 3: results for men

Figure S2. Age-specific estimates of smoking and quitting behaviours by socioeconomic position among men (≥16y) in Great Britain. Lines represent modelled weighted prevalence by age modelled non-linearly using restricted cubic splines (see **Supplementary File 1** for details of model selection) and occupational social grade (ABC1 = more advantaged; C2DE = less advantaged). Shaded bands represent 95% confidence intervals. Bars represent unmodelled weighted prevalence estimates within age bands (also presented with 95% CIs in **Table S10**). Unweighted sample sizes by age are provided in **Table S5**.

Additional file 3: results for men

Table S7. Modelled age-specific estimates of smoking prevalence and the quit attempt rate by socioeconomic position among men (≥16y) in Great Britain

Occupational social grade[3]	Smoking prevalence[1] % [95% CI] ABC1	C2DE	PR [95% CI][4]	Quit attempt rate[2] % [95% CI] ABC1	C2DE	PR [95% CI][4]
Age (years)[5]						
16	22.3 [19.7–25.0]	22.0 [18.6–25.9]	0.99 [0.92–1.26]	46.6 [41.4–51.9]	56.8 [50.0–63.3]	1.22 [1.06–1.51]
20	21.5 [19.8–23.3]	25.6 [23.0–28.5]	1.19 [1.07–1.38]	45.3 [41.2–49.5]	53.1 [47.5–58.5]	1.17 [1.05–1.40]
25	20.5 [19.1–21.9]	30.0 [27.8–32.3]	1.47 [1.28–1.56]	43.7 [40.6–46.8]	48.4 [44.3–52.6]	1.11 [1.02–1.29]
30	19.2 [17.7–20.8]	33.3 [30.5–36.3]	1.74 [1.50–1.75]	41.9 [39.4–44.5]	44.0 [40.7–47.4]	1.05 [0.97–1.19]
35	17.6 [16.1–19.2]	34.6 [31.5–37.8]	1.97 [1.69–1.96]	40.0 [37.4–42.7]	39.9 [36.9–43.1]	1.00 [0.91–1.13]
40	15.7 [14.6–16.8]	33.2 [30.7–35.8]	2.12 [1.85–2.17]	37.9 [35.1–40.9]	36.3 [33.1–39.6]	0.96 [0.85–1.10]
45	13.8 [13.0–14.6]	30.0 [28.2–31.9]	2.18 [1.96–2.33]	35.6 [32.6–38.8]	33.1 [29.8–36.6]	0.93 [0.81–1.09]
50	12.2 [11.4–13.0]	26.3 [24.6–28.0]	2.16 [2.01–2.42]	33.1 [30.1–36.2]	30.6 [27.4–34.1]	0.92 [0.79–1.08]
55	11.0 [10.2–11.8]	22.8 [21.2–24.4]	2.08 [1.98–2.38]	30.3 [27.6–33.2]	28.7 [25.7–31.8]	0.95 [0.81–1.09]
60	10.1 [9.3–10.9]	19.7 [18.1–21.3]	1.95 [1.82–2.23]	27.3 [24.8–29.9]	27.3 [24.6–30.1]	1.00 [0.84–1.15]
65	9.1 [8.2–10.0]	16.3 [14.6–18.1]	1.80 [1.54–2.05]	24.2 [21.7–27.0]	26.4 [23.7–29.2]	1.09 [0.90–1.26]
70	7.7 [6.9–8.5]	12.3 [11.0–13.9]	1.61 [1.21–1.82]	21.2 [18.1–24.6]	25.8 [22.4–29.5]	1.22 [0.93–1.50]
75	5.9 [5.2–6.7]	8.1 [6.9–9.5]	1.39 [0.89–1.58]	18.3 [14.6–22.7]	25.5 [20.9–30.7]	1.39 [0.98–1.90]
80	4.1 [3.1–5.3]	4.7 [3.3–6.6]	1.15 [0.62–1.33]	15.6 [11.4–21.0]	25.4 [19.4–32.6]	1.63 [1.01–2.55]

CI, confidence interval. PR, prevalence ratio.

[1] Among men.

[2] Among men who smoked in the past year.

[3] Occupational social grades ABC1 = more advantaged, C2DE = less advantaged.

[4] Prevalence ratio calculated as prevalence among less advantaged men divided by prevalence among more advantaged men, with 95% CIs calculated using bootstrapping (1,000 replications).

[5] Predicted weighted estimates for individual years of age from logistic regression models with age modelled using restricted cubic splines (with three knots; see **Supplementary File 1** for details of model selection). Note that the models used to derive these estimates included data from participants of all ages. Unmodelled weighted estimates within age bands are provided in **Table S10**.

Additional file 3: results for men

Table S8. Modelled age-specific estimates of the success rate of quit attempts and overall quit rate by socioeconomic position among men (≥16y) in Great Britain

Occupational social grade[3]	Success rate of quit attemptS5 % [95% CI] ABC1	Success rate of quit attemptS5 % [95% CI] C2DE	Success rate of quit attemptS5 PR [95% CI][4]	Overall quit rate[2] % [95% CI] ABC1	Overall quit rate[2] % [95% CI] C2DE	Overall quit rate[2] PR [95% CI][4]
Age (years)[5]						
16	22.7 [17.0–29.7]	27.9 [19.2–38.6]	1.23 [0.74–1.96]	15.2 [12.0–19.1]	18.1 [13.2–24.4]	1.19 [0.64–2.64]
20	25.2 [20.1–31.0]	26.3 [19.4–34.6]	1.05 [0.69–1.49]	16.1 [13.4–19.3]	16.5 [12.7–21.2]	1.03 [0.66–1.75]
25	28.3 [24.1–33.0]	24.5 [19.4–30.5]	0.86 [0.62–1.13]	17.1 [14.9–19.6]	14.8 [12.0–18.0]	0.86 [0.64–1.21]
30	31.5 [27.6–35.6]	23.0 [18.8–27.7]	0.73 [0.55–0.89]	18.0 [16.0–20.2]	13.2 [11.1–15.7]	0.74 [0.55–1.05]
35	34.2 [30.0–38.8]	21.7 [17.8–26.3]	0.63 [0.47–0.78]	18.5 [16.3–20.9]	12.0 [10.1–14.2]	0.65 [0.47–0.94]
40	36.4 [31.5–41.6]	20.9 [16.6–25.9]	0.57 [0.40–0.72]	18.5 [16.1–21.2]	11.0 [9.1–13.3]	0.60 [0.46–0.83]
45	37.7 [32.2–43.5]	20.5 [15.9–26.0]	0.54 [0.36–0.70]	17.8 [15.3–20.7]	10.3 [8.4–12.7]	0.58 [0.44–0.76]
50	37.9 [32.3–43.9]	20.6 [15.8–26.4]	0.54 [0.36–0.70]	16.4 [14.1–19.1]	9.9 [8.1–12.2]	0.61 [0.42–0.79]
55	36.9 [31.6–42.4]	21.3 [16.5–27.0]	0.58 [0.39–0.74]	14.4 [12.4–16.6]	9.9 [8.1–12.0]	0.69 [0.42–0.81]
60	34.5 [29.7–39.7]	22.6 [18.1–27.9]	0.66 [0.46–0.84]	11.8 [10.1–13.8]	10.1 [8.4–12.0]	0.85 [0.45–0.89]
65	31.2 [26.2–36.7]	24.7 [20.2–29.7]	0.79 [0.57–1.02]	9.2 [7.4–11.5]	10.5 [8.8–12.6]	1.14 [0.49–1.14]
70	27.2 [21.2–34.3]	27.3 [22.2–33.2]	1.00 [0.69–1.42]	6.8 [4.9–9.5]	11.3 [8.9–14.2]	1.65 [0.64–1.69]
75	23.1 [15.9–32.2]	30.6 [23.4–38.9]	1.33 [0.81–2.24]	4.9 [3.0–7.8]	12.3 [8.8–17.0]	2.52 [1.07–3.20]
80	19.0 [11.1–30.4]	34.5 [24.1–46.6]	1.82 [0.96–3.72]	3.4 [1.8–6.3]	13.5 [8.5–20.8]	4.02 [1.43–10.12]

CI, confidence interval. PR, prevalence ratio.

[1] Among men who tried to quit smoking in the past year.

[2] Among men who smoked in the past year.

[3] Occupational social grades ABC1 = more advantaged, C2DE = less advantaged.

[4] Prevalence ratio calculated as prevalence among less advantaged men divided by prevalence among more advantaged men, with 95% CIs calculated using bootstrapping (1,000 replications).

[5] Predicted weighted estimates for individual years of age from logistic regression models with age modelled using restricted cubic splines (with three knots for the success rate of quit attempts and five knots for the overall quit rate; see **Supplementary File 1** for details of model selection). Note that the models used to derive these estimates included data from participants of all ages.

Unmodelled weighted estimates within age bands are provided in **Table S10**.

Additional file 3: results for men

Table S9. Observed age-specific estimates of smoking and quitting behaviours among men (≥16y) in Great Britain

	% [95% CI]			
	**Smoking prevalence**[1]	**Quit attempt rate**[2]	**Success rate of quit attempts**[3]	**Overall quit rate**[2]
Age (years)[4]				
16-19	20.8 [18.0–23.5]	52.6 [45.5–59.8]	25.0 [15.7–34.3]	11.9 [6.9–16.9]
20-24	23.9 [21.8–26.0]	47.3 [42.5–52.1]	25.7 [19.6–31.9]	16.7 [13.2–20.2]
25-29	24.2 [21.8–26.6]	44.4 [39.1–49.7]	28.9 [21.6–36.3]	16.0 [12.1–19.9]
30-34	25.8 [23.7–28.0]	39.4 [34.9–43.8]	27.5 [21.4–33.5]	16.7 [13.4–20.0]
35-39	20.6 [18.5–22.8]	37.4 [32.1–42.8]	22.9 [15.5–30.4]	13.4 [9.7–17.1]
40-44	18.1 [16.3–19.9]	32.1 [27.1–37.1]	36.2 [27.1–45.4]	14.2 [10.5–17.8]
45-49	19.2 [17.0–21.3]	29.7 [24.1–35.2]	16.4 [9.2–23.7]	6.7 [3.9–9.4]
50-54	16.7 [15.1–18.3]	31.3 [26.5–36.0]	28.9 [20.6–37.1]	13.1 [9.8–16.4]
55-59	17.0 [15.3–18.6]	30.8 [26.0–35.5]	25.9 [17.7–34.0]	11.4 [8.1–14.6]
60-64	13.9 [12.4–15.4]	25.0 [20.1–29.9]	27.3 [17.5–37.1]	8.3 [5.5–11.2]
65-69	11.8 [10.3–13.2]	27.0 [21.3–32.6]	31.1 [19.6–42.7]	11.5 [7.3–15.7]
70-74	9.5 [8.2–10.9]	20.6 [14.4–26.8]	33.1 [16.9–49.2]	7.4 [3.2–11.6]
75-79	6.7 [5.5–7.9]	24.6 [17.0–32.1]	21.3 [5.4–37.1]	11.7 [5.9–17.5]
≥80	5.6 [4.4–6.9]	12.3 [4.9–19.6]	35.5 [1.9–69.2]	10.5 [1.6–19.4]

CI, confidence interval.
[1] Among men.
[2] Among men who smoked in the past year.
[3] Among men who tried to quit smoking in the past year.
[4] Observed weighted estimates within age bands. Sample sizes are provided in **Table S5**; note small denominators for some estimates (e.g., quitting outcomes in older age bands).

Additional file 3: results for men

Table S10. Observed age-specific estimates of smoking and quitting behaviours by socioeconomic position among men (≥16y) in Great Britain

Occupational social grade[4]	Smoking prevalence[1] ABC1	Smoking prevalence[1] C2DE	Quit attempt rate[2] ABC1	Quit attempt rate[2] C2DE	Success rate of quit attempts[3] ABC1	Success rate of quit attempts[3] C2DE	Overall quit rate[2] ABC1	Overall quit rate[2] C2DE
	% [95% CI]							
Age (years)[5]								
16-19	19.2 [16.2–22.2]	22.6 [17.7–27.5]	44.9 [36.5–53.2]	59.8 [48.6–71.1]	16.5 [7.3–25.7]	30.9 [16.8–45.0]	8.7 [4.2–13.1]	14.9 [6.2–23.7]
20-24	22.3 [20.0–24.7]	25.4 [21.9–28.9]	45.9 [40.3–51.4]	48.5 [41.1–56.0]	24.6 [17.7–31.6]	26.6 [17.1–36.1]	17.9 [13.7–22.2]	15.7 [10.3–21.1]
25-29	19.6 [17.1–22.0]	29.8 [25.4–34.1]	41.0 [34.8–47.2]	47.2 [38.9–55.4]	36.4 [27.0–45.7]	23.6 [13.0–34.3]	17.3 [12.7–21.9]	15.0 [9.0–21.0]
30-34	19.3 [17.3–21.4]	34.7 [30.6–38.8]	38.4 [33.2–43.7]	40.1 [33.3–47.0]	41.0 [32.6–49.5]	16.5 [8.5–24.5]	21.0 [16.7–25.3]	13.1 [8.2–18.0]
35-39	14.1 [12.1–16.1]	29.1 [25.0–33.2]	39.5 [32.4–46.5]	36.0 [28.5–43.6]	30.7 [19.9–41.4]	17.3 [7.4–27.3]	15.7 [10.6–20.9]	11.9 [6.8–17.0]
40-44	12.5 [10.8–14.2]	26.8 [22.9–30.6]	36.4 [30.0–42.7]	28.5 [21.1–35.9]	38.5 [27.6–49.4]	33.9 [19.0–48.7]	19.3 [14.0–24.5]	10.1 [5.1–15.1]
45-49	12.2 [10.4–14.1]	29.7 [25.3–34.0]	32.4 [24.9–39.9]	27.9 [20.1–35.6]	19.8 [8.9–30.7]	13.8 [4.2–23.4]	8.4 [4.1–12.7]	5.5 [2.0–9.1]
50-54	12.1 [10.5–13.7]	22.8 [19.7–25.9]	30.3 [24.4–36.3]	32.0 [25.0–39.0]	34.6 [23.4–45.9]	24.9 [13.3–36.4]	14.3 [9.7–18.9]	12.2 [7.5–16.8]
55-59	11.2 [9.6–12.7]	23.7 [20.7–26.7]	28.0 [21.6–34.4]	32.3 [25.8–38.8]	30.1 [17.3–43.0]	23.9 [13.6–34.1]	12.6 [7.7–17.5]	10.7 [6.4–15.0]
60-64	9.7 [8.3–11.1]	18.3 [15.7–21.0]	22.4 [16.2–28.5]	26.5 [19.6–33.3]	34.5 [19.1–49.9]	23.8 [11.5–36.1]	9.4 [5.0–13.8]	7.7 [4.0–11.4]
65-69	8.5 [7.1–9.8]	15.6 [12.9–18.3]	25.5 [18.1–32.9]	27.8 [19.9–35.7]	20.9 [7.0–34.8]	36.6 [20.7–52.5]	8.0 [3.5–12.6]	13.6 [7.5–19.6]
70-74	7.1 [5.8–8.5]	12.4 [9.8–14.9]	16.2 [8.5–24.0]	23.3 [14.5–32.1]	14.8 [0.0–33.9]	41.0 [19.9–62.1]	1.5 [0.0–3.7]	11.0 [4.4–17.6]
75-79	5.2 [4.0–6.4]	8.4 [6.3–10.5]	23 [13.4–32.6]	25.7 [14.8–36.6]	22.5 [4.7–40.2]	20.5 [0.0–43.8]	10.2 [3.3–17.1]	12.7 [4.2–21.3]
≥80	5.2 [3.8–6.7]	6.1 [4.0–8.2]	11.6 [2.4–20.9]	12.9 [1.4–24.3]	35.3 [0.0–78.8]	35.7 [0.0–86.3]	4.9 [0.0–11.5]	15.6 [0.3–30.9]

CI, confidence interval.

[1] Among men.

[2] Among men who smoked in the past year.

[3] Among men who tried to quit smoking in the past year.

[4] Occupational social grades ABC1 = more advantaged, C2DE = less advantaged.

[5] Observed weighted estimates within age bands. Sample sizes are provided in **Table S5**; note small denominators for some estimates (e.g., quitting outcomes in older age bands).
